# Task shifting healthcare services in the post-COVID world: A scoping review

**DOI:** 10.1371/journal.pgph.0001712

**Published:** 2023-12-08

**Authors:** Shukanto Das, Liz Grant, Genevie Fernandes

**Affiliations:** Usher Institute, University of Edinburgh, Edinburgh, United Kingdom; UP Manila: University of the Philippines Manila, PHILIPPINES

## Abstract

Task shifting (TS) is the redistribution of healthcare services from specialised to less-qualified providers. Need for TS was intensified during COVID-19. We explore what impact TS had on service delivery during the pandemic and examine how the pandemic affected TS strategies globally. We searched five databases in October 2022, namely Medline, CINAHL Plus, Elsevier, Global Health and Google Scholar. 35 citations were selected following the PRISMA-ScR guidelines. We analysed data thematically and utilised the WHO health systems framework and emergent themes to frame findings. We uncovered instances of TS in countries across all income levels. 63% (n = 22) of the articles discussed the impact of TS on healthcare services. These encompassed services related to mental healthcare, HIV, sexual and reproductive health, nutrition and rheumatoid diseases. The remaining 37% (n = 13) focused on how the pandemic altered strategies for TS, particularly in services related to mental healthcare, HIV, hypertension, diabetes and emergency care. We also found that studies differed in how they reported TS, with majority using terms “task shifting”, followed by “task sharing”, “task shifting and sharing” and “task delegation”. Our analysis demonstrates that TS had a substantial impact across healthcare systems. Modifying roles through training and collaboration strengthened workforce and enhanced diagnostic services. Strategic leadership played a crucial role in the process. More research on the financial aspects of TS during pandemics is required. Stakeholders generally accepted TS, but transferring staff between healthcare programs caused unintended disruptions. The pandemic reshaped TS, moving training, patient care and consultations to digital platforms. Virtual interventions showed promise, but digital access remained a challenge. Healthcare organisations adapted by modifying procedures, pathways and staff precautions. We recommend refining strategies for TS, and expanding on it to address workforce shortages, improve access, and enhance services, not only during crises but also beyond.

## Background

Shortage of skilled human resources for healthcare (HRH) makes healthcare systems and services vulnerable to fragmentation. Projections of global HRH deficits by the year 2035 range between 12.9–18 million [1,2]. Changing demographics and disease patterns, burdens of non-communicable diseases and emerging infections demand a larger qualified workforce. Given the correlation of provider numbers to service access, countries must instate appropriate ratios of skilled providers to population through workforce development [3]. Coronavirus disease (COVID-19) impacted services worldwide and has slowed progress towards sustainable development goal–3 of achieving ‘healthy lives and well-being for all’ [4,5]. Countries diverted finances, infrastructure and HRH towards caring for SARS-CoV-2 cases. Although resource-constrained settings were disproportionately impacted, the pandemic also affected nations with richer densities of facilities and HRH [6]. A survey across 105 World Health Organization (WHO) member nations reported that nine among 10 countries experienced major disruptions in essential healthcare services, further aggravating disease burdens of pre-COVID eras [7]. For instance, prevalence of anxiety and depression increased by 25%, with young people and women hit the worst [8]. Increase in depressive disorders costed over 49 million disability-adjusted life years [9]. Mortality due to human immunodeficiency virus (HIV) infections, tuberculosis and malaria are modelled to shoot by 10%, 20% and 36% respectively over the next five years [10].

Healthcare services can be improved by promoting innovation in diagnosis and treatment, especially by expanding on task shifting and task sharing (TS/S) [11]. TS/S are two approaches that move certain tasks which are typically performed by highly trained professionals, such as doctors, onto other providers and caregivers who have lesser training, such as nurses or community healthcare workers (CHWs) [12]. Often used synonymously, TS/S have differences. While task shifting (TS) focuses on providers assuming new roles through delegation from one hierarchy to other, task sharing is not as territorial and allows providers to expand duties by collaborating [13–15]. Both approaches remove bottlenecks in service provision in resource-limited settings by engaging existing HRH efficiently or by creating new cadres suitable to perform tasks [13,16]. TS/S should ideally be achieved by characterising tasks to be shifted or shared, subjecting new cadres to competency-based trainings and through continuous supervision and evaluation. This ensures that delegation is safe and effective, and the care delivered is of high quality [16]. TS/S has been used as pragmatic responses to low provider availability and acute demands. Chinese barefoot doctors [17], Russian Feldshers [18] and French *Officiers de Santé* [19] are historical examples whereby preventive care, diagnostics, treatment and emergency care have been delivered by less-specialised but trained caregivers in lieu of specialised providers. TS/S has been used in surgery, obstetrics, anaesthesia and ophthalmic procedures [20,21]. Experiences with HIV in Africa, led to classification of TS into five types, each involving distinct providers and functions [22]. Extensive work in this context subsequently led to the development of WHO global guidelines and recommendations on investing in TS/S, enabling policies and facilitating implementation of TS through service reorganisation [16].

The pandemic has exacerbated challenges posed by workforce shortages. Assessing impacts of TS/S during this crisis can offer insights into workforce strengthening strategies. This is particularly relevant as public health, humanitarian and climate-related emergencies are anticipated to occur more frequently, raising the question whether TS/S can be used as a tool in addressing such situations in the future. Few reviews have analysed how TS/S can benefit health systems beyond the pandemic. For instance, shifting psychotherapy to trained non-specialists can increase timely access to mental healthcare [23–25]. Integrating TS into hypertension, diabetes and obstructive lung diseases management models could preserve care continuum during future pandemics [26]. Task sharing family planning services with CHWs can lessen pregnancy-related mortality and child morbidity [27]. Human papillomavirus screening and vaccine education through CHWs could help fight cervical cancer [28]. While there exists literature on TS/S with respect to individual diseases or health issues based out of one or few comparable contexts, to our best knowledge our review is the first to study TS as a tool in its own right, specifically in light of a global pandemic.

We visualise (A) the impact of TS in healthcare services delivery since the onset of COVID-19 and (B) the impact the pandemic has had on strategies used to implementing TS; both at the global setting. We assess the former using the WHO health systems framework [29]. We chose the WHO health systems framework as it provides an internationally recognised comprehensive structure for assessing health system performance. This approach enabled us to understand how TS benefited service delivery, the workforce, information systems, access to medicines and technology, financing mechanisms and governance; all vital aspects to fathom the overall effectiveness of different countries’ responses to the pandemic and to identify areas which need reinforcement. Indeed, studies have applied the framework to evaluate impacts of public health emergencies on health systems previously. For example, during the Ebola outbreak in West Africa, researchers used this framework to conclude that efficient workforces are crucial for swift outbreak responses and that good-quality services relies heavily on the success of other blocks, such as proper financing and effective leadership and governance [30]. Similarly, we have applied the framework in context of COVID-19 to investigate how the sudden surge in cases strained systems and HRH worldwide. And then we continue on to shed light on how implementation strategies of TS evolved because of the pandemic.

## Methodology

**Fig 1:**
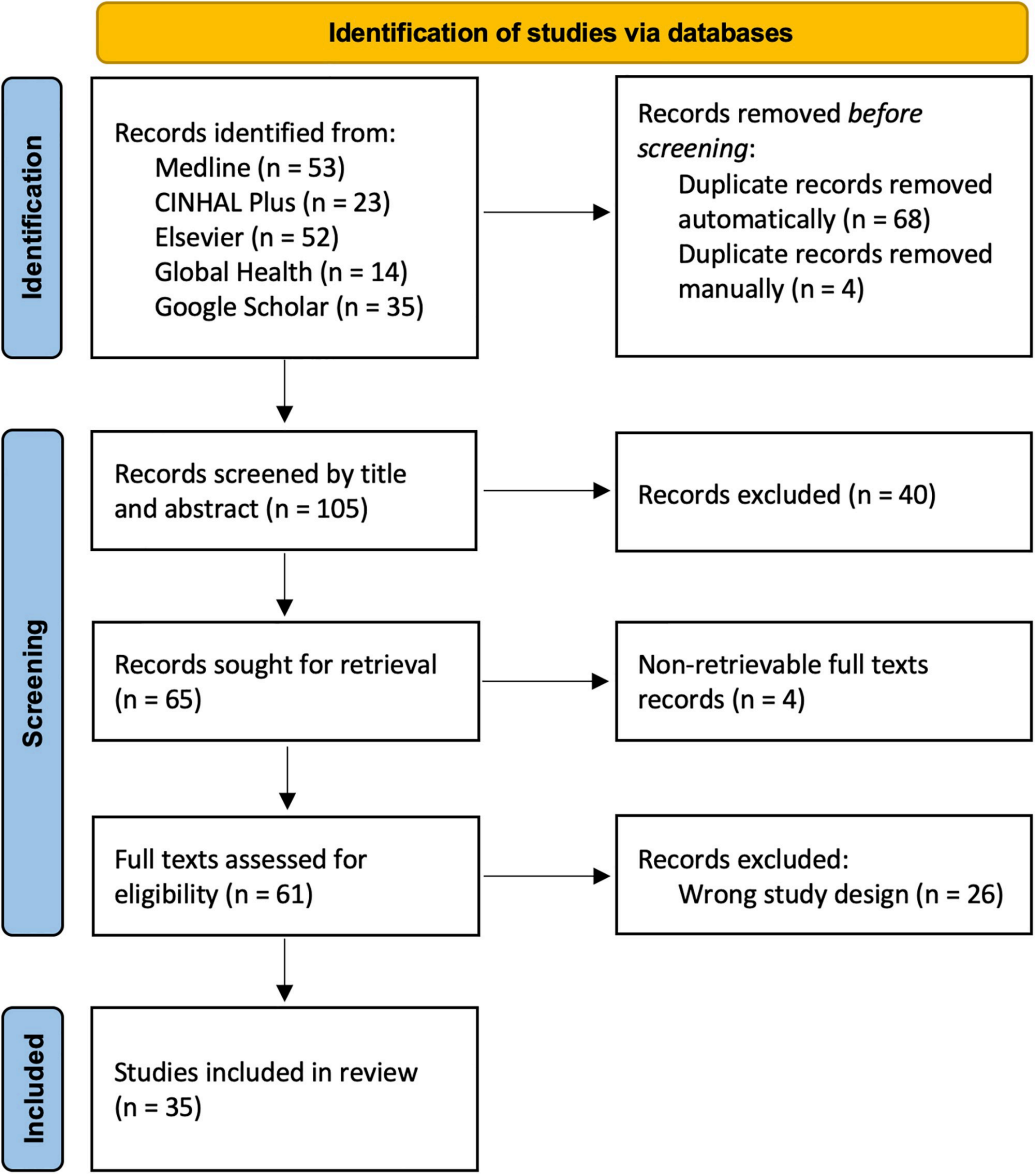
PRSIMA-ScR flow diagram of search and selection process of citations.

**Fig 2:**
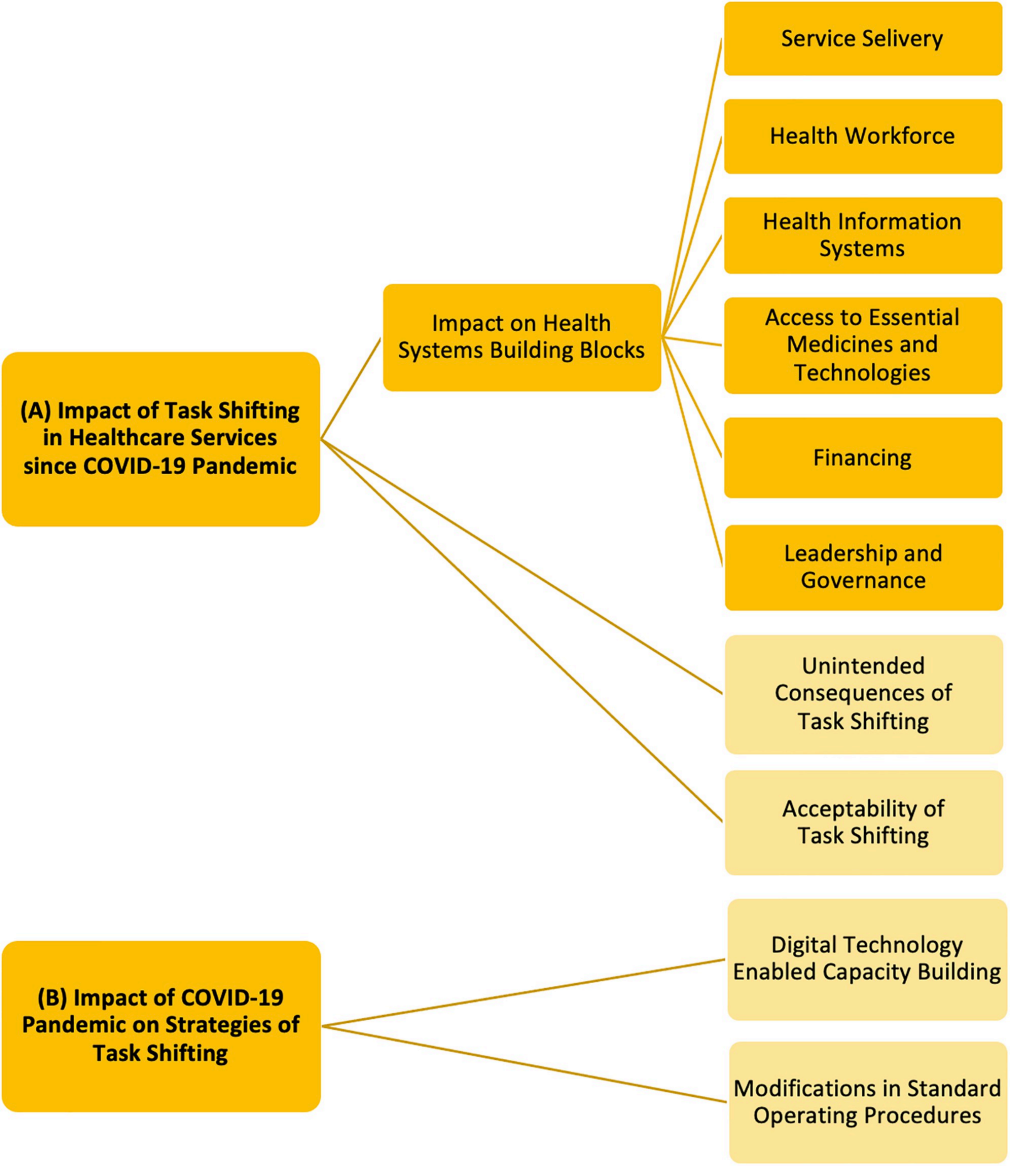
Coding frames used to undertake thematic analysis. Deductive themes represented in deep yellow colour. Inductive themes represented in light yellow colour.

Our scoping review aimed to explore two questions: (A) What impact has TS had in healthcare services globally since the onset of COVID-19 pandemic? (B) How has the pandemic impacted strategies of implementing TS worldwide? We used the following words and variations thereof to develop a search query: (“task shifting” OR “task sharing” OR “task shifting and task sharing” OR "task transfer" OR "task delegation” OR “TS/S”) AND (“healthcare” OR “health” OR “healthcare services”) AND (“COVID-19” OR "coronavirus” OR "covid-19 pandemic"). Search query was run across five databases in October 2022, namely Medline, CINAHL Plus, Elsevier, Global Health and Google Scholar. 177 citations were found and imported onto Covidence software [31] for screening. Duplications were removed. Records were first screened by titles and abstracts. Irrelevant articles were excluded. Full texts of relevant articles were sought and assessed. To examine eligibility, we applied a structured ‘Population, Concept and Context’ framework [32]. We included articles concerning any type of healthcare providers and caregivers (population). We included articles if they discussed TS from more to less specialised HRH (content). We included articles from all countries, spanning from onset of the COVID-19 pandemic in November 2019 (context). Only citations in English were included. Reviews, commentaries and theses were excluded. PRSIMA-ScR guidelines [33] were followed to screen and report citations (Fig 1). SD performed the initial screening. LG supervised the screening to ensure accuracy and consistency. Any discrepancies that arose during screening were addressed through discussions. 35 citations were selected in our final review. We also conducted a citation search to identify supplementary articles. However, no additional articles were found. From the final list of citations, we extracted key information, such as aim of study, terminology used to describe TS, country or context of study, study design, health conditions addressed, services shifted or shared, reasons for TS, cadres of HRH tasks moved from and moved onto, training provided to enable TS, results and conclusions of the study, and author recommendations. We analysed data thematically [34] using a deductive and inductive hybrid approach [35]. To investigate question (A), we began with a deductive phase. We used six predefined categories based on the building blocks of the WHO health systems framework [29]. We complemented this with an inductive phase, allowing us to explore additional emergent perspectives, further adding two more themes. In investigating question (B), two themes emerged inductively. Fig 2 depicts the coding tree used for analysis. The iterative process of combining deductive and inductive approaches proved to be effective in uncovering both structured and unforeseen insights. Detailed search strategy and protocol is included as S1 File.

## Results

**Fig 3:**
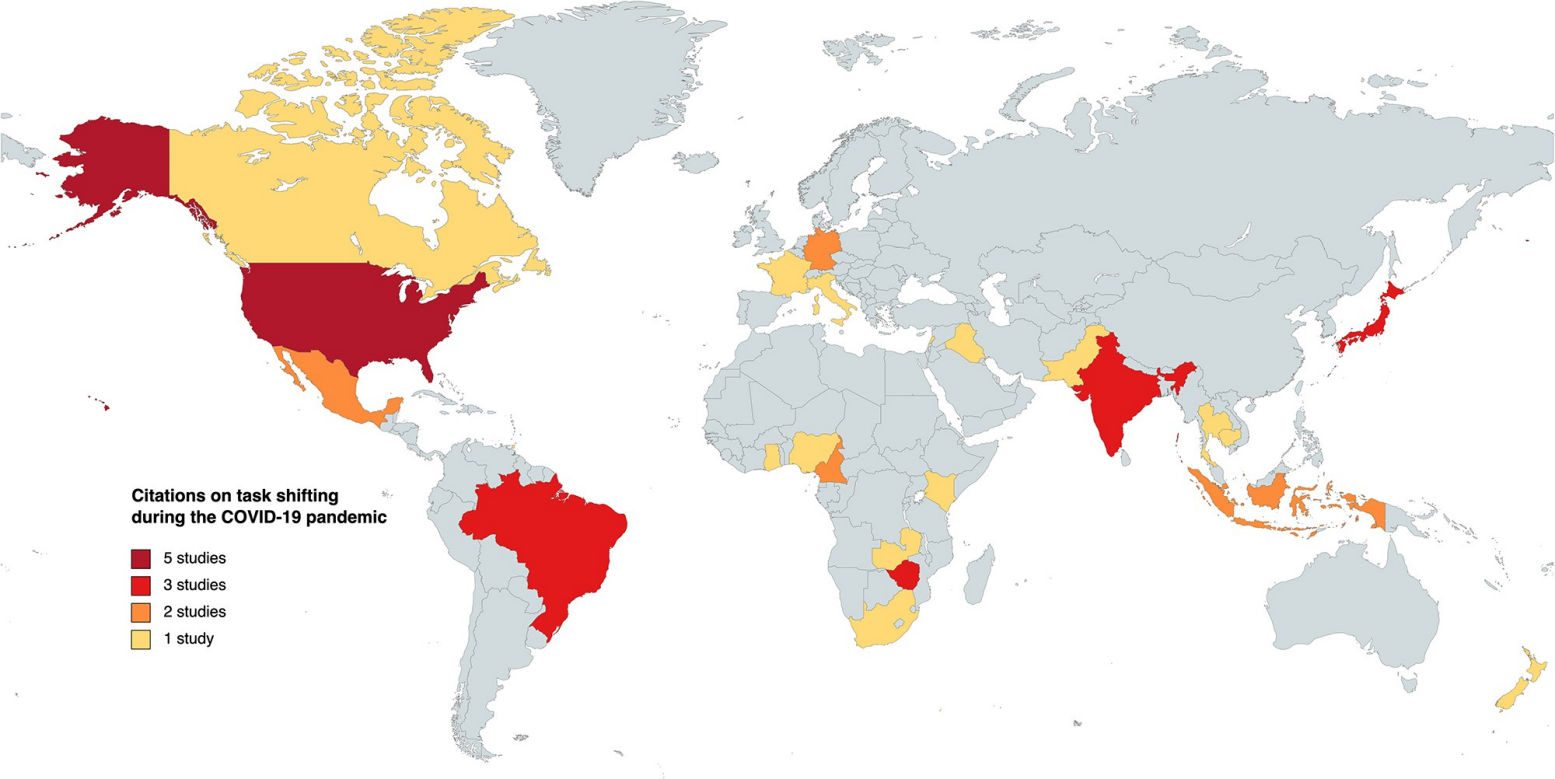
Global distribution of task shifting in healthcare services delivery since the onset of COVID-19 pandemic.

Our search covered articles discussing the impact of TS in health services since the pandemic across various domains. We found that 63% of the citations explored how TS affected health services, encompassing areas such as COVID-19 care (n = 14), mental healthcare (n = 2), HIV (n = 3), sexual and reproductive health (n = 1), nutrition (n = 1) and rheumatoid diseases (n = 1). The remaining 37% examined how the pandemic influenced strategies for TS, focusing on services related to mental healthcare (n = 7), HIV (n = 3), chronic illnesses including hypertension and diabetes (n = 2), alternative care modalities (n = 1) and emergency care (n = 1). These studies were drawn from a set of 25 countries, categorised based on their income levels: high-income countries (n = 9), upper middle-income countries (n = 5), low- and middle-income countries (n = 9) and low-income countries (n = 2). We visualised this distribution on a global map (Fig 3). An interesting finding was the variation in terminology used to describe the phenomenon of TS from specialised to less-qualified providers. We identified that 71.43% of the articles employed the term "task shifting" (n = 25), while 20.0% used "task sharing" (n = 6), 5.71% combined "task shifting and sharing" (n = 3) and 2.86% mentioned "task delegation" (n = 1). Table 1 presents our findings organised as evidence of the impact of TS on healthcare services worldwide since the onset of the pandemic. Table 2 outlines the impact of the pandemic on the strategies employed for implementing TS.

**Table 1:** Examples of task shifting in healthcare services delivery since COVID-19 pandemic.

Study	Country of study	Tasks shifted	HRH delivering services	Key findings
**COVID-19 testing, surveillance, communication and care management**				
Zafar, S *et al* [36]	Pakistan	Testing, contact tracing and risk communication	Lady Health Workers and Dengue Outreach Workers (CHWs)	COVID-19 surveillance hubs were established and CHWs were trained briefly before being deployed into communities As HRH were moved to surveillance, maternal health services and immunisation programs were ignored Need to improve information systems and TS during emergencies was highlighted
Gudza-Mugabe, M *et al* [37]	Zimbabwe	Testing, contact tracing and reporting	Laboratory technologists	HRH were trained in antigen rapid diagnostic test protocols and testing was decentralised from 1 to over 1000 centres Access to and uptake of testing increased Turnaround time of reporting reduced to less than a day Staff burnouts decreased
Honda, C *et al* [38]	Japan	Contact tracing, reporting and hospital coordination	Part time public health nurses (PHN)	Acute workloads caused full time PHN fatigue, anxiety and confusion at public health centres Full time PHN shifted workloads of epidemiological surveys, patient hospitalisation coordination, health monitoring of patient contacts and consultation to residents to part time PHNs as a measure to prevent burnouts and organisational dysfunction
Chidavaenzi, NZ *et al* [39]	USA	Testing, contact tracing, reporting and isolating	Public health staff at health department, Staff at detention centre and casino (non-healthcare staff)	Training in rapid antigen-based testing led to 3834 tests for 716 people Serial testing by non-health staff contributed to reduced case transmissions (only 1 positive case was recorded)
Raskin, SE *et al* [40]	USA	Testing, contact tracing, reporting and telephone-based risk communication	Dental assistants and dental hygienists	Clinic closures and acute demands pulled HRH into surveillance and community mobilisation Practices experienced significant call offs from assistants, hygienists and front desk operators, which burdened HRH further
Mohammed, A *et al* [41]	Nigeria	In-patient COVID care and other essential services	Medical students	80.4% students surveyed had good knowledge of COVID-19 78.3% students felt at risk of infections, yet 93% of them expressed willingness to assist care provision. Parental disapproval and fear were cited by those unwilling to get involved. Male respondents were more willing to assist, in comparison to females
Taylor, MK *et al* [42]	Multi-country (Singapore, Trinidad and Tobago, Iraq, India USA, Brazil and more)	Testing, contact tracing, triaging, risk communication and in-patient COVID care	Primary care physicians, medical students	Primary care physicians tested and triaged COVID-19 patients Primary care physicians increased awareness about social distancing, symptoms and quarantining Primary care physicians helped treat cases in intensive care units Primary care physicians undertook nursing procedures Final year medical students were involved in in-patient care
Eggleton, K *et al* [43]	New Zealand	Telephone triaging and nursing care	Nurses and practice receptionists	Receptionists were upskilled in telephone triaging and they determined whether patients needed General physician (GP) consultations or referrals Nurses and receptionist teams ran separate respiratory units to isolate patients with possible symptoms Routine clinical tasks of COVID-19 were shifted onto nurses TS increased workloads for nurses and receptionists, but freed GPs to attend more patients TS improved system efficiency and outputs
Yoshioka-Maeda, K *et al*[44]	Japan	Telephone consultations	Office support staff (non-healthcare staff)	Telephone consultations included queries on prevention measures and patient flow pathways; which did not need PHN involvement Telephone consultations can be shifted onto low-level staff and office workers through training, creation of script-based manuals and monitoring
Yoshioka-Maeda, K [45]	Japan	Telephone consultations, information management, resource management	Office support staff and external company (non-healthcare staff)	PHN developed a response manual for telephone consultations on COVID-19 and trained teams of office support staff to handle routine queries of local residents PHN created a web-based system to host patient information management and shifted clerical work to office support staff PHN shifted personal protective equipment management to external inventory management companies, increasing available nursing time and allocative efficiency
Helmi, M *et al*[46]	Indonesia	Intensive care services	General practitioners and medical students	ICUs reported inadequate availability of equipment, service support on call specialists and ineffective TS, leadership and communication among hospital staff Shifting services from specialists to general practitioners and students needs to be supplemented with training on roles, rights, communication and management skills
Sono-Setati, ME *et al* [47]	South Africa	Clinical auditing and resource management	Hospital staff (not specific)	Sub-standard management of COVID-19 cases, medical records and resources and staff anxiety, confusion and stress was reported Hospitals should expand roles of HRH to include TS to address staff shortages and burnouts Clinical audits should be conducted and reviewed routinely
Köppen, J *et al*[48]	Germany	Emergency procedures for infection control	Emergency paramedics	Although federal infection control law authorised TS, TS and skill-mixing was not mentioned in state policies directly Only Saxony-Anhalt specified the law and provided information supplementing the federal law Emergency paramedics were allowed to perform certain medical tasks provided they had competencies relevant to standard operating procedures and treatment pathways Medical directors of services needed to enlist tasks shifted and ensure adequate documentation
Faria de Moura Villela, E *et al* [49]	Brazil	In-patient COVID care and emergency medical services	Physicians, nurses and hospital staff	TS of services was seen in COVID wards (39.1%), intensive care units (11.6%) and emergency departments (15.9%) Shifts in roles and corresponding salary cuts coincided with reporting of anxiety and depression in HRH
**Mental health screening and therapeutic interventions**				
Ortega, AC *et al* [50]	Mexico	Psychosocial support, psychological first aid, grief management and palliative mental health services	Primary care physicians, community healthcare workers and non-clinical office staff	Non-profit Compañeros En Salud trained non-specialist providers on stress, anxiety and depression screening, identifying nonverbal cues, referral systems, psychological first aid and self-care strategies and provided them with pocket field guides for reference HRH were trained in contact tracing and their home-visit questionnaires had questions on patient mood, anxiety and suicidal thoughts Alongside psychological support, they provided mental healthcare for patients who were grieving and patients in need of palliative mental healthcare due to COVID-19
Mukhsam, MH *et al* [51]	Malaysia	Psychosocial support	Medical students	400 international medical students were tested and quarantined upon return to the University from China Disinfection and decontamination team performed cleaning upon positive case contact Quarantine team isolated cases under investigations Mobile medical and promotion team led surveillance and health education Students were grouped in tens on online messaging platforms and were supervised by local mandarin-speaking peers, one in each group Supervisors provided psychosocial support and health education Supervisors ensured students filled home-monitoring questionnaires
**HIV consultation, testing, counselling and treatment services**				
Omam, LA *et al* [52]	Cameroon	Counselling, testing, patient follow ups, Antiretroviral therapy (ART) initiation and refilling	Primary care physicians, nurses and non-clinical staff	Primary care-based differentiated service delivery of ART to internally displaced people during COVID-19 was achieved via mobile clinics In 7 months, 14,623 persons were sensitised and 1,979 were tested, from which 122 tested positive and 33 placed on ART 28 loss-to-follow up patents were relinked to treatment and 209 consultations were conducted Mobile clinics were resource-effective in improving access of HIV services in conflict regions, but the model needs economic evaluation
Pry, J.M. *et al* [53]	Zambia	ART initiation and refilling, community mobilisation	CHWs	Health ministry COVID-19 mitigation guidelines for HIV recommended dispensing 6 multi-month ART to patients and using TS to communicate and mobilise patients to collect ART refills early Adjusted prevalence ratio (4.63; 95% CI 4.45 to 4.82) of early returns to collect ART improved significantly post guideline implementation Weekly receipt of 6 multi-month dispensation increased from 47.9% before to 73.4% post guideline implementation Proportion of late visits fell from 18.8% to 15.1% post guideline implementation
Abraham, SA *et al* [54]	Ghana	Prescribing and dispensing ART	Nurses	Placing nurses at pharmacies and TS prescribing and dispensing of ART onto them reduced patient clustering and expedited service delivery during COVID-19 pandemic Staff rosters and roles were changed to complement TS
**Others: Sexual and reproductive health; Nutrition; Rheumatoid diseases**				
Jacobi, L *et al* [55]	USA	Provision of contraceptives and dispensing medicines	CHWs	Informants reported that the pandemic has ‘primed’ stakeholders for TS and sharing services to and with community health workers at primary care level TS and sharing provision of contraceptives and dispensing medicines a ‘necessity’ in times of COVID-19 in conflict affected regions
Davis, C. *et al* [56]	Singapore	Behaviour contracts for nutrition	General physicians and nurses	In-patient services of a tertiary hospital underwent reorganisation of HRH, limiting teams to one physician, nutritionist and speciality nurse Behaviour contracts for patients and caregivers, charted by psychologists ordinarily, were being drawn up by physicians and nurses during COVID-19
Kuhlmann, E *et al* [57]	Germany	Care for rheumatoid arthritis and other inflammatory musculoskeletal disorders	General practitioners, rheumatology specialist assistants and other medical assistants	67% rheumatologists delegated tasks to rheumatology specialist assistants and other medical assistants Rheumatologists perceived TS to rheumatology specialist assistants (87%) and GPs (33%) as an efficient approach to address rheumatologist shortages 81% found cooperation with medical assistants and nurses as good or very good, while cooperation with GPs scored significantly lower

**Table 2:** Examples of the impact of the pandemic on strategies for task shifting.

Study	Country of study	Task shifted	HRH delivering services	Key findings
**Mental health screening and therapeutic interventions**				
Singla, DR *et al* [58]	Multi-country (USA and Canada)	Screening and therapy for perinatal anxiety and depression	Nurses and midwives	SUMMIT trial consists of 6–8 weekly sessions on behavioural activation (BA) therapy offered by trained HRH in-person or online HRH are trained in 4-day courses and monitored during 8-week internships Trial is comparing effectiveness of task sharing among telemedicine specialist, telemedicine non-specialist, in-person specialist and in-person non-specialist Lockdowns led training, supervision, data collection and follow-ups shift online; effectiveness of telemedicine-supported BA is being evaluated
Scazufca, M *et al* [59]	Brazil	Screening, referring and therapy for depression	CHWs	PROACTIVE is a task-shared psychosocial care intervention for depression in adults aged 60 and more, based on psychoeducation and BA CHWs receive 3 days of training and weekly supervision from clinicians COVID-19 alerted trial implementation Recruitment of new participants ceased Participants who had not completed all sessions were offered 2 telephone sessions substituting in-person programme sessions 8 month and 12 month follow up was done via telephone 62·5% participants in the intervention showed recovery from depression compared to 44·0% in the control group (adjusted odds ratio 2·16 [95% CI 1·47–3·18; p<0·0001]
Jordans, MJD *et al* [60]	Lebanon	Psychological intervention for children with severe emotional distress	CHWs	Proof-of-concept study evaluated outcomes of competency-driven training (CDT) in improving facilitator quality CDT used pre-training and in-training competency assessment outcome scores to adjust dosage of training, focus on competencies and feedback Lockdowns led training of facilitators move online CDT improved training outcomes by 18% without extending class duration
Dambi, J *et al* [61]	Zimbabwe	Screening and therapy for depression	Grandmothers	Physical distancing measures led Friendship Bench to shift onto WhatsApp and telephone to deliver therapy and communicate with patients Friendship Bench is piloting Inuka, a chat-based application, to further access and enhance help-seeking behavior A quasi-experimental RCT found Iuka to be feasible and demonstrated a decline in common depressive disorders, depression and anxiety and increase in health-related quality of life Connectivity, app instability, expensive mobile data and power outages were discovered as barriers to scaling up
Nirisha, PL *et al* [62]	India	Screening and referring patients with alcohol use disorders and mental health for depression	Accredited social health activists (ASHA, who are CHWs)	COVID-19 restrictions led trainings adopt hybrid mode 1-day in-person training method vs 1 day in-person training and digitally-driven 7 online longitudinal training was compared in a trial; screen positives and KAP scores were measured Online trained ASHAs identified significantly higher number of persons with potential alcohol use disorders [83%; p ≤ 0.001] and common mental disorders [4%; p = 0.018], while in-person trained ASHA identified significantly higher number of those with potential severe mental disorders [61.61%; p ≤ 0.001] Mean KAP score increased from 16.76 to 18.57 (p < 0·01) in ASHA mentored online and from 18.65 to 18.84 (p = 0.76) in in-person trained
Philip, S *et al* [63]	India	Screening and referring patients with substance abuse and mental health for depression	Primary care physicians	114 primary care doctors were trained, monitored and mentored virtually Post training case-bases scenario examinations reported 37% improvements in knowledge scores 80.7% and 47.7% trainees felt confident in identifying mental health issues in patients and their caregivers respectively 52.6% felt they understand when to refer to higher centres 60.5% felt confident in prescribing and managing patients with mental health issues 64.9% respondents felt confident in providing deaddiction services
Rodriguez-Cuevas, F *et al* [64]	Mexico	Psychosocial support, psychological first aid and grief management	Primary care physicians, community healthcare workers, community mental healthcare workers and non-clinical office staff	Non-profit Compañeros En Salud (CES) trained non-specialist providers on mental health screening, therapy and referrals Increase in number of people faced with stress and anxiety due to COVID-19 led CES’s intervention to reach COVID patients and families To mitigate infection transmission risks to CHWs, undertook home visits using PPE such as three-layered fabric masks, hand sanitisers (regular visits); N95 or KN95 respirator mask, face shield, isolation gown and gloves (visiting suspected had COVID-19 patients) They maintained 1.5 metres distance from patients They met at private ventilated areas other than inside the house, e.g. garden, patio or clinic’s backyard
**HIV consultation, testing, counselling and treatment services**				
Coulaud, P *et al* [65]	Cameroon	Consultation, counselling and ART	Nurses	Small district hospitals designated to provide HIV services had limited resources and inadequate number of doctors Hospitals with limited HRH and not practicing task-shifting, reported higher HIV transmission risk and ARV stock-outs Tasks shifting is recommended to maintain ART services delivery as financial resources is being increasingly diverted to pandemic relief
Lujintanon, S *et al* [66]	Thailand	ART initiation	Community healthcare workers	Implementation trial is engaging community-based organisations and key population lay providers to start same-day initiation of ART delivery Lay CHWs are trained in counselling, testing, PrEP, PEP and ART care Clients will be offered teleconsultation with physicians and ART home delivery during the COVID-19 pandemic Success of this trial will help overcome geographical and HRH barriers
Roche, SD *et al* [67]	Kenya	Consultation, counselling and ART delivery	Hospital staff (not specific)	One-stop shop (OSS) model at HIV clinics allowed relocation of drugs, equipment, patient files and PrEP services at one point of patient contact TS dispensing drugs to lower cadres of care reduced provider movement and patient wait times However, social distancing protocols caused proximal testing points to shut and few to transform into isolation centres OSS moved to farther community clinics; patient preferences were unmet Staff had to travel between centres; wasting productivity
**Others: Chronic illnesses including Hypertension, Diabetes; Emergency medical services**				
Kamvura, TT *et al* [68]	Zimbabwe	Screening of Hypertension and diabetes	Nurses and community health workers (grandmothers)	The Friendship Bench-based intervention is shifting screening of hypertension and diabetes screening onto trained HRH During lockdowns, grandmothers, nurses and other stakeholders used WhatsApp to disseminate knowledge, advise patients and track referrals
Oikonomidi, T *et al* [69]	France	Consultations and prescribing drugs for chronic illnesses	Digital technology-supported provision of care	Patients preferred teleconsultations for half of their future consultations Patients would use online symptom-checkers over contacting doctors for 22.0% of new symptoms Patients preferred remote monitoring instead of consultations for 52.3% of their treatment adaptations Prescription renewal and addressing acute or minor complaints were reported as circumstances considered appropriate Patients expressed that they seek quality assurance and supervision of results by doctors
Iwamoto, A *et al* [70]	Cambodia	Emergency bag-and-mask ventilation, incubator-side tube feeding and temperature measurement	Family caregivers (Fathers and grandmothers)	Asymptomatic infected family members entering neonatal care units risk transmitting COVID infections to patients and staff Thermal scanning and frequent hand hygiene were implemented Implementing a second line of screening through questions on symptoms and contact tracing is recommended Risk of transmitting infections necessitates hiring more nursing staff and adequate training and reducing reliance on family caregivers

We now present our findings grouped under the specific 10 analytical themes discussed previously in the methodology section.

### Service delivery

To combat the surge in SARS-CoV-2 cases, governments focused on epidemiological surveys. In Arizona, USA, a large-scale rapid antigen-based serial screening initiative involved non-clinical staff from local health departments and detention centres. They were trained in sample collection, reporting, confidentiality and administration. Over 28 days, they conducted 3,834 tests on 716 individuals, resulting in only one positive case. Despite factors like community immunity, vaccinations and social distancing, this TS significantly reduced transmission [39]. The pandemic also heightened the need for mental healthcare. Mexican organisation Compañeros En Salud and the Chiapas health ministry trained primary care physicians, CHWs and non-clinical staff in psychological first aid and referrals. These HRH received pocket guides to offer psychosocial support to the community. CHWs included anxiety and suicidal thought assessments in home-visit questionnaires. They cared for grieving patients and referred severe cases to higher-level care. This approach expanded care coverage, addressing stressors stemming from the pandemic and offering solutions to fill service gaps in resource-limited settings [50].

### Health workforce

During the pandemic, roles were reassigned to meet increased demands. For instance, in the USA, primary care physicians shifted to intensive care, while in Brazil, final year medical students assisted with SARS-CoV-2 cases. In Singapore, tasks traditionally performed by psychologists, such as drafting nutrition behaviour contracts for patients and caregivers, were taken on by physicians and nurses [42]. Increased workloads among public health nurses (PHN) in Tokyo caused anxiety, frustration and fatigue, leading to task shifts to part-time PHN, other nursing staff and support personnel [38]. Teleconsultations by PHN during the first wave focused on prevention measures and referral pathways, prompting managers to shift these tasks to lower-level staff and office workers using scripted manuals. This allowed PHN to concentrate on infection control and management [44]. In Ghana, the central teaching hospital adapted staff roles and schedules to transfer medication prescribing and ART dispensing from pharmacists to nurses, ensuring ART continuity and reducing patient waiting times at HIV clinics [54]. While TS typically refers to transfer of responsibilities from more to less specialised providers, the pandemic saw instances where highly-qualified providers assumed roles traditionally performed by less-qualified counterparts. Primary care physicians conducted fieldwork to screen, test and triage patients in Singapore, Trinidad and Tobago, and raised awareness about social distancing, symptoms and quarantine in Iraq and India. In Italy, a shortage of nursing staff led primary care physicians to perform nursing procedures and therapies [42]. Dental hygienists from dental clinics were redirected towards community screening and telehealth services to educate community members [40].

### Health information systems

Sub-standard medical record management during the pandemic resulted in clinical auditing system failures. A multi-hospital study in South Africa recommended TS data collection and audit reporting from clinical staff, particularly emergency healthcare providers, to other HRH to enhance care and coordination. This shift helped reduce mortality, morbidity, stress and burnouts [47]. Japanese PHN moved teleconsultations to office staff and developed web-based data systems for exchanging COVID-19 patient information. They also shifted hospital coordination, clerical tasks and inventory management to external companies, which saved nursing time, reduced workloads and improved supply allocation efficiency [45].

### Access to essential medicines and technologies

Centralised testing, limited HRH and logistical challenges created difficulties in meeting testing demands. To enhance access, expand testing capacity and accelerate reporting, Zimbabwe shifted antigen-based diagnostics testing to laboratory technicians. These technicians received training in testing techniques, simultaneous to upgrading testing equipment with COVID-specific software. By TS testing onto laboratory technicians, Zimbabwe decentralised testing from a single facility to over a thousand centres, increasing testing availability and uptake and reduced report turnaround time to less than a day and mitigated staff burnout [37].

### Financing

Our search did not yield economic evaluations of TS since the pandemic. Nevertheless, one article, which discusses the successful decentralisation of ART in Cameroon, highlights the value of economic evaluations for TS-based models. The intervention involved establishing mobile units and transferring testing, counselling, ART initiation, refilling and follow-up to non-clinical staff within primary care and community-based facilities. While the intervention enhanced service access, particularly in conflict-affected regions, the authors underscores the importance of conducting economic evaluations for TS-based models before considering their expansion into other hard-to-reach settings [52].

### Leadership and governance

Timely preparedness plans and implementation aided pandemic management. For instance, a case study examined the German Federal and State policies regarding TS [48]. While Federal policies recommended TS, states did not fully incorporate these recommendations. The Federal infection control law authorised TS from doctors to paramedics, emphasising their competencies, non-involvement with severe patients and documentation. However, the law lacked specifics. Only Saxony-Anhalt’s state policies elaborated on these competencies, aligning them with treatment protocols and documentation tools. The lack of comprehensive guidance hindered nationwide TS adoption. Foresighted direction at all organisational levels was key. Hospitals acknowledged the necessity for TS in intensive care units (ICUs), recognising resource constraints. An evaluation in Indonesia identified inadequacies in space, equipment and specialist distribution. To address these, leaderships initiated TS within ICUs through training on rights, responsibilities, communication, coordination and incentives for providers [46]. At the University Malaysia Sabah in Borneo, strategies were implemented to mitigate COVID-19’s impact, with a focus on mental health through TS. Stringent policies on screening, personal protective equipment (PPE) and attendance were enforced, supported by dedicated teams for surveillance, health promotion, quarantining and sanitation. In a unique approach, local medical students proficient in Mandarin supervised quarantined international Chinese students, providing psychosocial support and health education. These actions prevented COVID-19 importation into the campus [51]. Another case study on Zambia’s COVID-19 mitigation guidelines for HIV demonstrated that swift policy implementation reduced interruptions in ART delivery. The government’s recommendations included dispensing six multi-month ART supplies to patients and TS for patient communication and mobilisation through trained lay providers. This increased early ART collections, with a more than fourfold rise in weekly multi-month dispensations and a reduction in late visits [53].

### Unintended consequences of task shifting

TS also resulted in unintended consequences. Government-employed CHWs in Islamabad were trained for surveillance and deployed to identify SARS-CoV-2 cases and bring patients for testing. However, this led to them abandoning their other engagements within maternal health and immunisation programs; creating service gaps [36]. In Brazil, about three-quarters of doctors, nurses and hospital staff experienced structural changes and role shifts. Most TS occurred in COVID-19 wards, ICUs and emergency departments. These role changes often coincided with salary cuts and increased levels of anxiety and depression among staff [49]. The pandemic also transformed doctor-patient relationships and the nature of practice teams. In New Zealand, receptionists at General Practitioners (GPs) were upskilled in telephone triaging and routing patients to consultations, nursing care or other facilities. Nurses and receptionist teams ran separate respiratory units for triaging patients with symptoms to prevent transmission, and these patients were seen by GPs separately. While TS improved outputs and freed GPs to attend patients, managing different patient streams added to workloads of nurses and receptions, necessitating support staff hires [43].

### Acceptability of task shifting

TS, which is generally met by resistance from healthcare providers, was accepted during the pandemic. For example, the success of CHWs in contraceptives and medicine dispensing that improved family planning services access, made stakeholders view TS as necessary during workforce shortages [55]. In Germany, a shortage of rheumatologists led to care for rheumatoid arthritis and musculoskeletal disorders being delegated to GPs and rheumatology specialist assistants. In fact, rheumatologists found TS to specialist assistants more useful than delegating to GPs. Workforce shortages primed stakeholders toward TS, showcasing its feasibility and acceptability [57]. Surveys in Nigerian teaching hospitals assessed student knowledge and provider willingness for COVID care. About 90% of students expressed willingness to assist, although they had concerns about infections and parental disapproval [41]. TS onto medical students with suitable training effectively boosted service capacity.

### Digital technology enabled capacity building

COVID-based containments caused interventions cease routine activities and rapidly adopt new delivery models. SUMMIT trial is TS behavioural activation for perinatal depression and anxiety from psychologists, psychiatrists and social workers onto nurses and midwives [58]. Lockdowns in USA and Canada caused training and supervision of providers, follow-ups and data collection to migrate onto patient-centred virtual systems. Likewise, the PROACTIVE study, which assessed effectiveness of psychoeducation-based support for older adults delivered by trained CHWs with no higher education or formal training, had to resort to telephone-based care due to lockdowns in Brazil. This transition led to a higher recovery rate of 62.5% among participants receiving virtual care, compared to 44% among those receiving care as usual [59]. Another study from Lebanon demonstrated that online-delivered competency-driven training significantly improved facilitator abilities in providing psychological treatment to distressed adolescents by 18%, highlighting the potential of telemedicine-supported community-based mental health interventions [60]. Similarly, Friendship Bench, which shifts screening and psychological support for common mental disorders to trained grandmothers, adapted to digital platforms during lockdowns. A pilot study of their chat-based application in Zimbabwe found the implementation feasible and effective. Users experienced reduced depression and anxiety and improved quality of life [61]. The Friendship Bench model was also extended to include screening for comorbid conditions like diabetes and hypertension, maximising the benefits of TS [68].

Two Indian studies on substance use and addiction demonstrate how digital training improved mental health screening and provider competence. In one study, virtually trained CHWs identified significantly more alcoholism cases (83%) and showed improved knowledge, attitude and practice scores compared to in-classroom trained providers [62]. In another study, primary care physicians, virtually trained and monitored by specialists, saw a 37% increase in knowledge scores [63]. These results suggest the potential for online capacity building at the primary care level. Monitoring and physician consultations in the decentralisation of ART initiation onto trained lay providers in Bangkok also transitioned to virtual platforms. Insights from this TS-based trial can aid in addressing geographical and HRH challenges [66].

COVID-19 reshaped healthcare practices, emphasising on online health-seeking behaviour and delivery. A French survey assessed post-pandemic preferences, with a shift towards teleconsultations and symptom-checking applications over traditional physician-led methods. Notably, 22% of patients with chronic illnesses preferred online symptom-checkers for new symptoms. While many found digital TS suitable as pre-consultation tools, it was not found universally appropriate, especially for patients with anxiety or complex symptoms. Patients emphasised that TS to digital applications should involve accreditation by quality control authorities and physician supervision [69].

### Modifications in standard operating procedures

Organisations modified their standard operating procedures to protect staff during the pandemic. Organisations that deploy trained non-specialists to deliver mental health services, extended their scope to address pandemic-related stress and anxiety. Providers undertook patient-home visits outdoors while using extensive PPE [64]. Neonatal ICUs in Cambodia shift incubator-side tube feeding and bag-and-mask resuscitation onto fathers and grandmothers. Due to concerns about family transmission of SARS-CoV-2, thermal scanning, sanitation, symptom-based questionnaires and contact tracing were implemented as an additional measure. Facilities also hired and trained additional nurses to reduce reliance on TS with family caregivers, reversing some progress made with TS [70]. In Kenya, the one-stop shop model improved PrEP delivery, uptake and continuation. By relocating client files, equipment and drugs to one-stop shops and delegating PrEP dispensing to lower cadres, staff movement and waiting times were reduced. However, increasing caseloads and social distancing measures led to transforming of one-stop shops into isolation centres and the relocation of one-stop shop operations to distant community clinics, resulting in time and productivity losses [67]. Likewise, in Cameroon, district-level centres employed TS of ART prescription and administration onto nurses to maintain ART continuation [65].

## Discussion

### (A) Impact of task shifting on healthcare services globally since the onset of COVID-19 pandemic

The WHO’s early recommendation of TS/S as a response to COVID-19 set the stage for this crucial healthcare strategy. As our study reveals, TS was pivotal in addressing challenges posed by the pandemic. Delegation of COVID-19 screening and contact tracing to non-clinical staff helped reduce transmission rates [39], underlining the effectiveness of TS in dealing with such crises Furthermore, training of primary care physicians and CHWs to provide psychosocial and palliative support expanded the reach of mental health services during the pandemic, particularly in resource-constrained settings [50]. These innovative solutions not only tackled the immediate issues brought on by the pandemic but also exemplified the adaptability and resilience of TS in service delivery, providing valuable lessons for future crises. Interestingly, some scenarios saw more-qualified providers stepping into roles traditionally performed by less-qualified counterparts. For example, primary care physicians took on responsibilities such as screening, triaging and community education. They even performed nursing procedures [42]. The specific strategies employed to utilise the healthcare workforce varied among countries, but the common thread was the implementation of TS through rational reorganisation, cross-training and collaboration. This flexibility proved instrumental in managing the multifaceted challenges posed by COVID-19. This evidence demonstrates that TS is not only a practical approach to the current pandemic but also a vital component in building systems that can withstand the demands of future crises, ensuring continued access to essential services and adapting to shifting circumstances. It showcases how healthcare systems can optimise their available resources and enhance capacity to respond swiftly and effectively when faced with unforeseen challenges.

The pandemic revealed limitations in health information systems, particularly in managing medical records and conducting clinical audits [47]. This inadequacy prompted the adoption of TS as a strategic response to enhance the reliability and efficiency of these systems. The inclusion of non-clinical staff in data management, as demonstrated in Japan where PHN shifted teleconsultations to office staff, has proven to be a versatile solution that mitigates burnouts among emergency care providers, thus improving care coordination and reducing adverse outcomes [45]. This experience underscores the potential of leveraging technology to facilitate TS to optimise provider time and reduce workloads. The importance of rapid access to essential medicines and diagnostic tools became evident during the pandemic. Zimbabwe’s approach to addressing increased testing demands by upskilling laboratory technicians, thus expanding testing availability and reducing reporting times, exemplifies the power of TS [37]. The decentralisation of diagnostic care through TS, entrusting certain responsibilities to less-qualified but systematically trained providers, can improve access, expedite diagnosis and enhance the overall resilience of the system. However, it is crucial to emphasise that such an approach must be carefully planned, well-supervised and consider rigorous regulatory checks to ensure patient safety and maintain quality of care.

One gap in the literature is the absence of economic evaluations related to TS during the pandemic. Economic assessments are essential for comprehending the cost-effectiveness of TS-based models. These not only provide stakeholders with valuable insights into the financial implications of implementing TS, but also offer a foundation for evaluating sustainability and long-term benefits of such approaches. This highlights the need for future research to focus on economic evaluations, as these are crucial for decision-making and resource allocation.

Effective stewardship and regulatory policies were pivotal in guiding healthcare practices during the pandemic. The case of German Federal and State policies on TS highlights that lack of clarity and specificity in guidelines can result in inconsistent outcomes even across the same nation [48]. In contrast, quick policy interventions and effective delegation on part of a Bornean university showcased how key TS was in preventing SARS-CoV-2 case importations and achieving zero-COVID status on campus [51]. Similarly, hospital leaderships in Indonesia recognised deficiencies in ICUs in and shifted services through quick training programs and incentivisation [46]. Policy rooted in TS through lay providers substantially improved ART uptake during the pandemic [53]. Clear unambiguous policies and proactive decision-making across all organisational levels are necessary to lead to more consistent and successful implementation of TS, ultimately improving health outcomes during pandemics.

The unintended effects of TS during the pandemic bring to light the need for comprehensive planning and strategies. While TS effectively addressed acute healthcare needs, its implementation had repercussions on other essential services. For example, the deployment of CHWs for COVID-19 surveillance in Islamabad disrupted maternal health and immunisation programs [36], highlighting the necessity of considering the broader implications of TS on systems. This underscores the importance of a nuanced approach that optimises TS’s benefits while minimising unintended consequences. Structural changes brought about by TS led to increased anxiety and depression among providers [49]. This finding emphasises the need for support systems to address the mental health and well-being of HRH during times of crisis. A balanced strategy that takes into account both the positive impacts and potential drawbacks of TS is essential for sustainable healthcare delivery.

Experiences of TS in humanitarian settings [55] and with rheumatology specialist assistants in Germany [57] illustrate how the success of TS can alter perceptions among stakeholders, particularly when confronted with workforce shortages. The shift towards more open attitudes regarding TS as a means to ensure continued access to services during times of crisis highlights its feasibility and potential acceptability. Stakeholder engagement and transparent communication about the rationale behind TS can mitigate resistance and foster acceptance. Institutionalisation of TS can prove to be a valuable long-term strategy, not limited to pandemics but also for improving care access beyond crises. This approach aligns with the need for adaptable healthcare systems capable of responding effectively to various challenges while maintaining the quality of care.

### (B) The impact of pandemic on strategies used to implementing task shifting

The pandemic’s impact led to rapid adaptations in TS and digital technology emerged as a cornerstone of capacity building. Disruptions in routine activities prompted innovative delivery models. The SUMMIT [58] and PROACTIVE [59] trials exemplified the effectiveness of moving training, supervision and therapy provision to patient-centred virtual systems or telephones, leading to reduced depression and anxiety rates in patients. These findings underscore the potential of telemedicine-supported community mental health interventions in enhancing service quality and access.

The pandemic also ushered innovation in substance use and addiction treatment, demonstrating the potential of online-based capacity building to augment mental health screening and provider knowledge [62,63]. These results suggest the potential for online capacity building at the primary care level, offering efficiency and reducing infection risks, travel costs, and carbon emissions. However, challenges related to the accessibility and affordability of digital technology persist. Digital applications played a pivotal role in initiatives like the Friendship Bench [61], allowing them to reach users during lockdowns and effectively reduce depression and anxiety. However, challenges such as connectivity, expensive mobile data, and power outages need to be addressed to scale up these solutions. connectivity issues, application instability, expensive mobile data, and power outages [61].

The decentralisation of ART initiation to trained lay providers, with virtual monitoring and physician consultations, improved accessibility, particularly addressing geographical and human resource barriers [66]. The surge in telehealth utilisation and investments during the pandemic emphasises the importance of striking a balance between traditional and digital healthcare. Ensuring accreditation and quality control for digital pre-consultation tools is critical to maintain patient safety and appropriateness, especially for addressing anxiety and diverse symptoms. This highlights the need for a careful transition towards more digital healthcare solutions, maintaining a focus on quality and patient well-being. COVID-19 changed how healthcare was sought, delivered and regulated online. Telehealth utilisation increased 38 times and investments, tripled; fuelling innovation to improve access and affordability [71].

Healthcare organisations exhibited remarkable adaptability during the pandemic by modifying their standard operating procedures to implement TS effectively. These operational adjustments were vital not only for ensuring continued patient care but also for protecting their staff from potential COVID-19 transmission. The ability to flexibly embrace TS practices underscores healthcare organisations’ adaptability, particularly in the context of Human Resources for Health (HRH) shortages and financial constraints. The implementation of TS in Cameroon played a crucial role in enhancing the accessibility of Antiretroviral Therapy (ART) and helped address the HRH limitations associated with routine prescription and administration [65]. In Kenya, the introduction of One-Stop-Shop (OSS) models streamlined the delivery of PrEP but faced challenges due to pandemic-induced social distancing measures, leading to the need for increased travel between centres [67].

Moreover, healthcare organisations adapted to safeguard their HRH by expanding training in mental health support [50]. In this context, providers implemented stringent safety measures during home visits, including the use of Personal Protective Equipment (PPE) [64]. The Neonatal Intensive Care Units (ICUs) in Cambodia shifted responsibilities to reduce infection risks, emphasising the necessity of additional staff and training [70]. These examples not only showcase healthcare organisations’ resilience in the face of HRH constraints but also highlight the critical role of adaptability in ensuring continued healthcare services, particularly during crisis situations. While TS and procedural adaptations improved healthcare accessibility, it’s important to acknowledge that challenges emerged as the pandemic’s conditions continued to evolve. This underscores the need for continuous assessments and room for flexibility in organisational responses to address HRH limitations and maintain service quality during uncertain circumstances.

Lastly, it is extremely vital to discuss the observed variability in the terminology used to describe the phenomenon of TS from specialised to less-qualified providers. This variation hints at the potential for confusion and ambiguity, especially when researchers are attempting to synthesise new findings or when policies related to TS are under development and implementation. To address this, researchers, healthcare professionals, policy makers and other stakeholders should reach a consensus on the meanings and accepted terminology when studying and reporting on TS and task sharing, and the differences in each approach. Standardising the language used in this context can lead to clearer communication, stronger research and the development of well-informed policies in healthcare services delivery.

### Limitations and implications of future research

It is important to note that the evidence on TS we have synthesised is based on published literature that originates from a limited set of nations and does not include many low-income countries. This lack of representation can result in a geographical bias. Additionally, few citations we examined lacked specific information concerning which tasks were transferred and the particular HRH involved. Future studies should strive to provide a more detailed account of these. Furthermore, future research can delve into how TS guidelines are framed, and implemented, especially in emergency contexts. This examination can help refine policies and practices, making them more effective and adaptable in crises situation. Economic evaluations of TS during pandemics are another area of significant importance. Assessing cost-effectiveness and financial implications of TS could offer valuable insights for decision-makers. Therefore, future research should also place a specific focus on this aspect.

## Conclusions

In light of the ongoing global health challenges, the COVID-19 pandemic has shed light on the critical role of TS in maintaining the integrity of health services. Our review underscored the effectiveness and adaptability of TS in addressing needs during this unprecedented crisis. As the WHO acknowledged early on, the swift adoption of TS/S was imperative to respond to the mounting stresses. Our findings showcase that during the pandemic, TS was instrumental in bridging gaps in healthcare delivery across a spectrum of services, from mental health and substance use interventions to critical care in ICUs. The success stories spanning multiple countries demonstrate the versatility of TS, which ranged from reorganising roles among HRH to upskilling new providers. Such adaptability has been critical not only for addressing the immediate challenges presented by the pandemic but also for building resilience against future crises. However, it is essential to recognise that benefits of TS are often accompanied by unintended consequences, such as disruptions in non-COVID essential services and an increase in anxiety and depression among HRH. These findings emphasise the importance of comprehensive planning and mitigation of potential drawbacks when implementing TS. To harness the full potential of TS, healthcare systems must adopt a nuanced and balanced approach that maximises its benefits while minimising these side effects. In conclusion, our study reinforces the significance of TS in enhancing healthcare delivery during pandemics. It is imperative that organisations, policymakers and researchers globally acknowledge the potential of TS as a flexible dynamic approach for service delivery during emergencies. By doing so, we can not only respond more effectively to ongoing health crises but also build healthier futures.

## Supporting information

S1 File Protocol used to conduct literature search and data analysis. (DOCX)

S2 File PRISMA-ScR checklist for reporting data. (DOCX)
