# Supplementary material for: Task shifting healthcare services in the post-COVID world: A scoping review
Source: PLOS Glob Public Health. 2023 Dec 8;3(12):e0001712. doi: 10.1371/journal.pgph.0001712 (PMC10707657; doi:10.1371/journal.pgph.0001712)
Supplement: S1 File — (DOCX) [file pgph.0001712.s001.docx]

**Protocol for literature search and data analysis**

Das S, Grant L. ‘Task shifting healthcare services in the post-COVID world: A scoping review’

We conducted a scoping review as they help understand the range, scale and nature of a research theme, outline and present evidences, highlight gaps in literature and help evaluate whether undertaking a full systematic review pertaining to the question of interest would be beneficial [1,2]. Scoping reviews differ from systematic reviews in a way that the latter usually has well-defined questions and study designs to begin investigating literature with, while the former has a broader focus and can include diverse types of studies in it [2]. We follow the methodological framework of 5 stages [2] to conduct this scoping review:

**Stage 1: Identifying the research question**

Our aim has been to gather evidences from literature exploring two questions:

1. What impact has TS had in healthcare services globally since the onset of COVID-19 pandemic?
2. How has the pandemic impacted strategies of implementing TS worldwide?

**Stage 2: Identifying relevant studies**

An initial search was carried out on Medline via PubMed on October 07, 2022 using PubMed. This search returned 136 articles. But on screening titles and abstracts, over 1/2 citations were found irrelevant hinting at the need of refinement of query code.

Following this, the search strategy was revised, the search code was rewritten and nw run across a total of 5 databases between October 11-12, 2022:

1. Medline via PubMed (<https://pubmed.ncbi.nlm.nih.gov>)
2. CINHAL Plus via EBSCO (<https://www.ebsco.com/products/research-databases/cinahl-database>)
3. Elsevier via Scopus (<https://www.scopus.com>)
4. Global Health via OVID (<https://ovidsp.ovid.com>)
5. Google Scholar (<https://scholar.google.com>)

**Search terms for Medline PubMed is shown here as sample:**

(("healthcare"[Text Word] OR "health"[Text Word] OR "healthcare programs"[Text Word] OR "healthcare delivery"[Text Word] OR "health care organisation and systems"[Text Word] OR "health systems"[Text Word] OR "delivery of health care"[Text Word] OR "health plan implementation"[Text Word] OR "healthcare workforce"[Text Word] OR "healthcare provider*"[Text Word] OR "healthcare provider training*"[Text Word] OR "caregiver"[Text Word] OR "health service*"[Text Word] OR "service delivery"[Text Word] OR "health services use"[Text Word] OR "primary care"[Text Word] OR "community clinic"[Text Word] OR "rural health clinics"[Text Word] OR "rural health services"[Text Word] OR "primary care management"[Text Word] OR "patient care management"[Text Word] OR "primary care services"[Text Word] OR "healthcare providers"[Text Word] OR "models of care"[Text Word] OR "clinical management"[Text Word] OR "emergency care"[Text Word] OR "preventive care"[Text Word] OR "preventive healthcare"[Text Word] OR "preventive medicine"[Text Word] OR "public health"[Text Word] OR "health promotion"[Text Word] OR "hospitalization"[Text Word] OR "hospital care"[Text Word] OR "surger*"[Text Word] OR "surgical care"[Text Word] OR "palliative care"[Text Word] OR "rehabilitative care"[Text Word] OR "curative care"[Text Word] OR "patient-centered care"[Text Word] OR "patient centred-approach"[Text Word] OR "patient-centred care"[Text Word] OR "person-centred approach"[Text Word] OR "person-centred care"[Text Word] OR "human resources for health"[Text Word] OR "patient care team"[Text Word] OR "healthcare teams"[Text Word] OR "community health worker"[Text Word] OR "community health worker training*"[Text Word] OR "community nurs*"[Text Word] OR "ayush"[Text Word] OR "ayurveda"[Text Word] OR "homeopathy"[Text Word] OR "asha"[Text Word] OR "anganwadi"[Text Word] OR "medical informatics"[Text Word] OR "traditional medicine"[Text Word] OR "barefoot doctor*"[Text Word] OR "physician assistant*"[Text Word] OR "physician associate*"[Text Word] OR "non-physician clinicians"[Text Word] OR "mid level health worker*"[Text Word] OR ("Health Care Category"[MeSH Terms] OR "Occupational Groups"[MeSH Terms] OR "Disciplines and Occupations Category"[MeSH Terms] OR "analytical, diagnostic and therapeutic techniques and equipment category"[MeSH Terms] OR "Diseases Category"[MeSH Terms] OR "Psychiatry and Psychology Category"[MeSH Terms]) OR ("health policy"[Text Word] OR "healthcare policy"[Text Word] OR "policy implementation"[Text Word] OR "healthcare strategy"[Text Word] OR "universal health coverage"[Text Word] OR "health systems research"[Text Word] OR ("Public Policy"[MeSH Terms] OR "Organizational Policy"[MeSH Terms] OR "Diseases Category"[MeSH Terms])) OR ("quality of healthcare"[Text Word] OR "Quality of Health Care"[Text Word] OR "quality of care"[Text Word] OR "healthcare outcomes"[Text Word] OR "patient satisfaction"[Text Word] OR "universal health coverage"[Text Word] OR "access to care"[Text Word] OR "evidence-based treatment"[Text Word] OR "performance measures"[Text Word] OR "healthcare evaluation"[Text Word] OR "quality of life"[Text Word] OR "health services research"[Text Word] OR "health workforce development"[Text Word] OR "health workers planning"[Text Word] OR "health human resources planning"[Text Word] OR ("Quality of Health Care"[MeSH Terms] OR "Diseases Category"[MeSH Terms])) OR ("human capital"[Text Word] OR "healthcare investments"[Text Word] OR "health economics"[Text Word] OR "sustainable healthcare"[Text Word] OR "cost-benefit analysis"[Text Word] OR ("Health Care Economics and Organizations"[MeSH Terms] OR "Diseases Category"[MeSH Terms]))) AND ("task shift*"[Text Word] OR "task shift*"[Text Word] OR "TS/S"[Text Word] OR "task shar*"[Text Word] OR "task shar*"[Text Word] OR "task shifting and task sharing"[Text Word] OR "task transfer*"[All Fields] OR "task delegat*"[Text Word]) AND ("2019-ncov disease"[Text Word] OR "2019-ncov disease"[Text Word] OR "2019-ncov diseases"[Text Word] OR "covid"[Text Word] OR "covid19"[Text Word] OR "covid 19"[Text Word] OR "sarscov"[Text Word] OR "sars cov 2"[Text Word] OR "corona virus"[Text Word] OR "coronavirus"[Text Word] OR "coronavirus diseases"[Text Word] OR "novel coronavirus"[Text Word] OR "covid pandemic"[Text Word] OR "covid19 pandemic"[Text Word] OR "covid-19 pandemic"[Text Word] OR "sars-cov2 detection"[Text Word] OR "sars-cov-2 diagnosis"[Text Word] OR "long covid"[Text Word] OR "long covid19"[Text Word] OR "long covid-19"[Text Word] OR "post covid"[Text Word] OR "post covid19"[Text Word] OR "post covid-19"[Text Word] OR "covid management"[Text Word] OR "covid treatment"[Text Word] OR "covid management"[Text Word] OR "covid-19 management"[Text Word] OR "covid-19 clinical management"[Text Word] OR "covid treatment"[Text Word] OR "covid19 treatment"[Text Word] OR "covid-19 treatment"[Text Word] OR (("COVID-19 Testing"[MeSH Terms] OR "COVID-19 Serological Testing"[MeSH Terms] OR "covid 19"[MeSH Terms] OR "COVID-19 Vaccines"[MeSH Terms] OR "sars cov 2"[MeSH Terms] OR "post-acute COVID-19 syndrome"[Supplementary Concept] OR "adult multisystem inflammatory disease covid 19 related"[Supplementary Concept]) AND "pediatric multisystem inflammatory disease covid 19 related"[Supplementary Concept]))) AND ((fha[Filter]) AND (fft[Filter]))

Results of the search is shown as under:

| **Search Query** | **Databases** | **Medline**  11 Oct, 2022 | **CINHAL** 11 Oct, 2022 | **Elsevier**  12 Oct, 2022 | **Global Health**  12 Oct, 2022 | **Google Scholar**  12 Oct, 2022 |
| --- | --- | --- | --- | --- | --- | --- |
| **Healthcare**  (Healthcare delivery OR Healthcare policy OR Quality of healthcare OR Health economics) | | 28,164,950 | 2,362,831 | 9,069,952 | 1,310,086 | About 2,540,000 |
| **COVID-19** | | 314,238 | 271,712 | 437,661 | 97,191 | About 638,000 |
| **Task Shifting OR Task Sharing** | | 2,316 | 976 | 4,116 | 798 | About 17,300 |
| **(Task Shifting OR Task Sharing) AND Healthcare** | | 2,183 | 826 | 2,108 | 785 | 153 |
| **(Task Shifting OR Task Sharing) AND Healthcare AND COVID-19** | | 53 | 23 | 52 | 14 | 35 |
| **Total unique citations after removing duplications** | | | | | | **105** |

**Stage 3: Selecting studies**

Citations were imported into Covidence systematic review management software (<https://app.covidence.org>). Duplications were removed automatically and manually. Citations were screened as per the following selection criteria. PRSIMA-ScR guidelines [3] were followed to screen and report citations (see Fig 1 in the paper). **35 citations** were selected finally. Citation search was also conducted on the derived articles, but no other articles were found.

| **Inclusion criteria** | **Exclusion criteria** |
| --- | --- |
| - All articles on task shifting services from higher to lower professional cadres of healthcare providers and caregivers - All geopolitical contexts and care settings - All diseases, health conditions and healthcare problems - Citations published since the onset of COVID-19 pandemic, i.e. November 2019 - Citations available in English language only | - Articles on delegation of work between same cadres of healthcare providers and caregivers - Studies relating to task switching in cognitive science and learning - All literature reviews, commentaries, editorials, dissertations and conference proceedings |

**Stage 4: Charting of the data**

We read full-texts and extracted the following information from the identified citations independently:

- Title of the paper
- Author information
- Journal of publication
- Date of publication
- Aim of the study
- Focus on task shifting and/or task sharing
- Country of study
- Study design and tool
- Sampling frame
- Sample size
- Inclusion and exclusion criteria, if any
- Type of analysis
- Health condition(s) addressed
- Healthcare services shifted/shared
- Reason for task shifting and/or task sharing
- Cadres of workforces tasks moved from
- Cadres of workforces tasks moved to
- Training provided to enable task shifting and/or task sharing
- Duration of training provided to enable task shifting and/or task sharing
- Guidelines followed to enable task shifting and/or task sharing
- Results and conclusions of the study
- Recommendations of the author(s

**Stage 5: Collating, summarising and reporting results**

We analysed extracted data thematically [4], using a deductive and inductive hybrid approach [5] to coding data. For the first question “What role has task shifting played in healthcare services delivery since the COVID-19 pandemic?”, we used a top-down approach using the WHO Health Systems Framework [6] and checked for impact on each system building block, as well as simultaneous unintended consequences and general acceptability of task shifting. For the second question, “How has the COVID-19 pandemic impacted strategies of task shifting globally?”, we used a bottom up approach to analyse the dataset as per emergent themes. We used the coding tree depicted in Fig 2 in the paper. We discuss our findings in details in the paper. Deductive themes represented in deep yellow colour. Inductive themes represented in light yellow colour.

**References:**

1. Mays N, Roberts E, Popay J. Synthesising research evidence. Studying the organisation and delivery of health services: Research methods. 2001. p. 33. doi:10.4324/9780203481981

2. Arksey H, O’Malley L. Scoping studies: Towards a methodological framework. International Journal of Social Research Methodology: Theory and Practice. 2005;8(1): 19–32. doi:10.1080/1364557032000119616

3. Tricco AC, Lillie E, Zarin W, O’Brien KK, Colquhoun H, Levac D, et al. PRISMA extension for scoping reviews (PRISMA-ScR): Checklist and explanation. Ann Intern Med. 2018;169(7): 467–473. doi:10.7326/M18-0850

4. Braun V, Clarke V. Using thematic analysis in psychology. Qual Res Psychol. 2006;3: 77–101. doi:10.1191/1478088706qp063oa

5. Xu W, Zammit K. Applying Thematic Analysis to Education: A Hybrid Approach to Interpreting Data in Practitioner Research. Int J Qual Methods. 2020;19: 1–9. doi:10.1177/1609406920918810

6. World Health Organization. Everybody’s business: strengthening health systems to improve health outcomes: WHO’s framework for action. Production. 2007. [cited 2 December 2022]. Available from: https://iris.who.int/bitstream/handle/10665/43918/9789241596077_eng.pdf
